# Association Between Plasma Redox State/Mitochondria Function and a Flu-Like Syndrome/COVID-19 in the Elderly Admitted to a Long-Term Care Unit

**DOI:** 10.3389/fphys.2021.707587

**Published:** 2021-12-15

**Authors:** Elena Grossini, Diego Concina, Carmela Rinaldi, Sophia Russotto, Divya Garhwal, Patrizia Zeppegno, Carla Gramaglia, Seval Kul, Massimiliano Panella

**Affiliations:** ^1^Laboratory of Physiology, Department of Translational Medicine, University of Eastern Piedmont, Novara, Italy; ^2^AGING Project Unit, Department of Translational Medicine, University of Eastern Piedmont, Novara, Italy; ^3^Anteo Cooperativa Sociale Onlus, RSA Belletti Bona, Biella, Italy; ^4^Public Health, Department of Translational Medicine, University of Eastern Piedmont, Novara, Italy; ^5^Psychiatric Unit, Department of Translational Medicine, University of Eastern Piedmont, Novara, Italy; ^6^Department of Biostatistics, Faculty of Medicine, Gaziantep University, Gaziantep, Turkey

**Keywords:** aging, antioxidants, COVID-19, mitochondria, oxidative stress

## Abstract

**Background/Aims:** It is widely known that the imbalance between reactive oxygen species (ROS)/antioxidants and mitochondrial function could play a pivotal role in aging and in the physiopathology of viral infections. Here, we correlated the plasma oxidants/antioxidants levels of the elderly admitted to a long-term care (LTC) unit with clinical data in relation to flu-like disease/COVID-19. Moreover, *in vitro* we examined the effects of plasma on cell viability, ROS release and mitochondrial function.

**Materials and Methods:** In 60 patients admitted to LTC unit for at least 1 year at moderate or high care load, demographic and clinical variables were taken. Blood samples were collected for the evaluations of oxidants/antioxidants, as thiobarbituric acid reactive substances, 8-hydroxy-2-deoxyguanosine, 8-isoprostanes, superoxide dismutase activity, glutathione, and vitamin D. *In vitro*, human umbilical vascular endothelial cells (HUVEC) were used to examine the effects of plasma on viability, ROS release and mitochondrial membrane potential.

**Results:** The results obtained showed that the redox state of the elderly was quite balanced; mitochondrial membrane potential of HUVEC was reduced by about 20%, only. Also, the correlation analysis evidenced the association between mitochondrial function and the patients’ outcomes. Interestingly, lower levels of mitochondrial membrane potential were found in the elderly who had symptoms suggestive of COVID-19 or with a confirmed diagnosis of COVID-19.

**Conclusion:** The results of this study highlight the importance of mitochondrial function in the tendency to get a flu-like syndrome like COVID-19 in the elderly admitted to LTC unit. This information could have clinical implications for the management of old population.

## Introduction

Aging arises from the accumulation of oxidative damage to cells and tissues, which is associated with a progressive increase in the chance of morbidity and mortality (Junqueira et al., 2004). Hence, the redox state plays a crucial role in aging and in the development of age-related diseases and conditions (Liu et al., 2017).

The imbalance between peroxidative factors and reactive oxygen species (ROS) over antioxidants (Sies, 1985) would lead to oxidative damage of carbohydrates, lipids, proteins, mitochondria, as well as, DNA (Pacifici and Davies, 1991; Phaniendra et al., 2015), which, finally, could trigger the aging process by not clearly defined mechanisms (Belenguer-Varea et al., 2020).

Among various intracellular organelles, mitochondria, could play a crucial role in aging since any impairment of mitochondrial function could enhance vulnerability to oxidative stress (Eckmann et al., 2013; Chistiakov et al., 2014; Islam, 2017). In physiological conditions, the oxidants/antioxidants ratio, which is maintained by enzymatic and non-enzymatic systems, can finely regulate several cell functions. In the elderly, the efficiency of the endogenous antioxidant system often shows a decline, which could account for the high prevalence of cardiovascular and neurologic/psychiatric disorders (Corbi et al., 2008; Checconi et al., 2020).

Recent evidence showed that the redox state and the mitochondrial functions are also associated to the pathogenesis and severity of infectious diseases of viral origin, including COVID-19 (Ren et al., 2020; Dominguez et al., 2021). Even though COVID-19 can infect people of all ages, the elderly and co-morbid patients are at higher risk of having poor clinical outcomes and prognosis (Guan et al., 2020; Li et al., 2020; Panfoli, 2020; Yang et al., 2020).

It is noteworthy that at the basis of COVID-19 infections there would be excessive inflammation, activation of cytokines storm and increased ROS release (Checconi et al., 2020; Wang J.Z. et al., 2020), which may activate apoptosis of epithelial cells and endothelial cells and, subsequently, vascular leakage and abnormal immune system response leading to acute lung injury/acute respiratory distress syndrome or death (Channappanavar and Perlman, 2017).

Although certain levels of ROS are crucial for the modulation of immunological responses and for clearing viruses, excessive ROS, as above reported, can rapidly destroy not only virus-infected cells but also normal cells in lung and even heart, resulting in multiple organ failure. In addition, alterations between oxidants/antioxidants would facilitate specific steps of the virus lifecycle and activate an inflammatory response (Checconi et al., 2020). Therefore, the endogenous antioxidant system could play a crucial role and a potential anti-oxidative therapy could be proposed to face COVID-19 infection and improve organ function (Wang J.Z. et al., 2020; Zhang et al., 2020).

For all these reasons, the study of any possible association between the plasmatic levels of oxidants/antioxidants, mitochondrial function, and the onset of COVID-19 in the elderly, could be useful to better clarify both the role of the redox state in old people and its possible implications to their susceptibility to COVID-19, which could be relevant in the actual context of COVID-19 pandemic (Wang J.Z. et al., 2020).

Therefore, we performed a study aimed to evaluate the possible association between the redox state condition, measured as plasma levels of oxidants/antioxidants and as changes of ROS release and mitochondrial function of human umbilical vascular endothelial cells (HUVEC) treated with plasma, and the onset of COVID-19 in compromised elders.

## Materials and Methods

### Study Design and Setting

This study is a part of a larger cohort study aimed to identify possible predictors of longevity in elders admitted to long-term care (LTC)/nursing homes (Longevity Check Up – Long C-UP Study). The Long C-UP Study was approved on July 27th, 2019 by the Ethical Committee of the “Azienda Ospedaliera Maggiore della Carità” University Hospital in Novara, and registered (registration number: CE 31/19 approved). The study was performed in the “Belletti Bona” nursing home, a 120 beds LTC facility for elderly based in Biella, Piedmont Region Italy. Piedmont Region was at the epicentre of the spread of the COVID-19 pandemic in Italy.

### Sample Size and Patient’s Selection

The sample size was defined adopting the incidence of COVID-19 as the primary outcome (*p*-level one sided = 0.05). Because during the first wave of the pandemic in Italy, the rate of subjects with COVID-19 in nursing homes ranged between 30 and 50%, the calculation of the sample size was done assuming a proportion of 0.4. Considering testing for single proportions with a first type error of 5% (95% confidence intervals), a width of the confidence interval of 0.25, 60 subjects were enrolled (power = 0.80). We included consecutive patients that have been admitted to the “Belletti Bona” for at least 1 year with a moderate or high care load of care (Barthel Index – BI < 40) (Mahoney and Barthel, 1965; Granger et al., 1979) that were able to express their consent to the study. Patients with cognitive impairment were excluded from the study (Short Portable Mental Status Questionnaire – SPMSQ ≥3 errors) (Pfeiffer, 1975).

### Data Collection

Each patient gave signed informed consent for the handling of clinical data and for the use of plasma samples for experimental purposes, according to the Italian National Laws and Regulations (Legge 22 dicembre 2017, n. 219) and with The Code of Ethics of the World Medical Association (Declaration of Helsinki). To keep the anonymity of data collection and analysis an alphanumeric code was randomly assigned to each patient. The biological samples were collected at baseline in January 2020 (see below collection of sample paragraph). The baseline data also included patients’ demographic and clinical characteristics: gender (male/female) age (years), total BI (TBI) (score), SPMSQ (score), flu vaccination (yes/not). The clinical outcomes data were collected from clinical records using an anonymous data abstraction form throughout all the study period from February 1st to December 31st, 2020. Those included the onset of clinical COVID-19 syndrome (yes/not), the related therapy (yes/not), the confirmation of the COVID-19 disease with real-time reverse transcription polymerase chain reaction (rRT-PCR) test (yes/not) and the patient survival status (alive/deceased).

### COVID-19 Case Definition

Since rRT-PCR tests were not available until April 25th, 2020, we used a mixed approach to identify COVID-19 cases in our sample. First, from February to April 2020, we screened clinical records for symptoms suggestive of COVID-19. Accordingly, we defined as a clinical COVID-19 case each patient who presented at least one of the most common clinical features at the onset of illness. As it is reported in literature, these included: fever ≥ axillary temperature over 99.5°F/37.5°C, cough (dry or with expectoration), fatigue/asthenia/myalgias, dyspnoea, anosmia/ageusia, blood oxygen saturation ≤92%, rhinorrhoea/rhinosinusitis, and diarrhea (Chen et al., 2020; Guan et al., 2020; Huang et al., 2020; Wang D. et al., 2020; Xu et al., 2020). On April 25th, 2020, the COVID-19 RT-PCR test for the qualitative detection of nucleic acid from SARS-CoV-2 in nasopharyngeal or oropharyngeal swabs was adopted in Italian nursing homes as a 2-week screening program for elderly. Therefore, until the end of the study (December 31st, 2020) we defined as a COVID-19 case each patient who was positive to the COVID-19 RT-PCR test, independently of possible associated symptoms. The possible associated symptomatic therapy included: anti-inflammatory drugs (paracetamol, dexamethasone, and/or other corticosteroids), antibiotics (all the classes, respiratory drugs, and/or oxygen provision), and low molecular weight heparins and/or antiaggregant drugs.

### Collection of Samples

Blood samples were taken from each donor, in the morning in fasting conditions by using BD Vacutainer tubes (sodium heparin as anticoagulant) on January 15th, 20th, and 24th, 2020 (baseline, T0 samples). Each sample was immediately centrifuged by a refrigerated centrifuge (Eppendorf, mod. 5702 with rotor A-4-38) for 10 min, at 3100 rpm at 4°C. The plasma obtained was divided into five tubes that were stored at −80°C at the Physiology Laboratory of the University of East Piedmont and further used for the quantification of markers of redox state, as specified below and in Supplementary Material. Furthermore, plasma samples were used also to perform *in vitro* experiments on human vascular endothelial cells (HUVEC). As it was mentioned before, plasma samples were handled in anonymous conditions.

### Plasma Markers of Redox State Evaluation

At the Laboratory of Physiology, various measurements were performed by biotechnologists under the supervision of Prof. Elena Grossini. In particular, we measured plasma markers of lipidic peroxidation, determined as thiobarbituric acid reactive acid substances (TBARS) release, 8-hydroxy 2 deoxyguanosine (8 OH-2dG), 8-isoprostanes, superoxide dismutase (SOD) activity, glutathione (GSH), and 25(OH) vitamin D, through specific assay. Furthermore, we evaluated plasma levels of Thymosin β4. Each measurement was performed in duplicate by using a spectrophotometer (VICTOR™ X Multilabel Plate Reader).

### Glutathione Quantification

Plasma GSH measurement was performed by using the Glutathione Assay Kit (Cayman Chemical, Ann Arbor, MI, United States), as previously described (De Cillà et al., 2019; Farruggio et al., 2019; Grossini et al., 2020) and reported in Supplementary File 1. GSH was detected at excitation/emission wavelengths of 405−414 nm.

### Thiobarbituric Acid Reactive Acid Substances Quantification

Plasma TBARS were determined as malonyldialdeide (MDA) release. MDA measurement was performed by using the TBARS assay Kit (Cayman Chemical) (De Cillà et al., 2019; Surico et al., 2019; Grossini et al., 2020) and described in Supplementary File 2. MDA was detected at excitation/emission wavelengths of 530–540 nm.

### 8-Hydroxy 2 Deoxyguanosine Quantification

Plasma 8 OH-2dG measurement was performed by using the 8-hydroxy 2 deoxyguanosine ELISA Kit (Abcam, Cambridge, United Kingdom) (Surico et al., 2017, 2019; Grossini et al., 2018, 2020; De Cillà et al., 2019, 2020; Farruggio et al., 2019, 2020), as described in Supplementary File 3. The 8 OH-2dG was detected using a wavelength of 450 nm.

### 8-Isoprostanes Quantification

Plasma 8-isoprostanes (F2 isoprostanes) measurement was performed by using the 8-isoprostanes ELISA Kit (Abcam) (Surico et al., 2017, 2019; Grossini et al., 2018, 2020; De Cillà et al., 2019, 2020; Farruggio et al., 2019, 2020). The 8-isoprostanes were detected using a wavelength of 450 nm. The methods are described in Supplementary File 4.

### 25(OH) Vitamin D Quantification

Plasma 25(OH) vitamin D was measured by using the 25(OH) vitamin D ELISA Kit (Abcam) (Surico et al., 2017, 2019; Grossini et al., 2018, 2020; De Cillà et al., 2019, 2020; Farruggio et al., 2019, 2020), as described in Supplementary File 5. Plasma 25(OH) vitamin D was quantified using a wavelength of 405 nm.

### Superoxide Dismutase Activity

Total SOD activity was determined in plasma by using the Superoxide Dismutase Activity Assay Kit (Abcam) (Surico et al., 2017, 2019; Grossini et al., 2018, 2020; De Cillà et al., 2019, 2020; Farruggio et al., 2019, 2020) as described in Supplementary File 6 and the reading was performed with a wavelength of 450 nm.

### Thymosin β4

Thymosin β4 (Human TMSβ4) was measured by using the Thymosin beta 4 (Human TMSβ4) ELISA Kit (FineTest; Wuhan Fine Biotech Co., Wuhan, China) (Surico et al., 2017, 2019; Grossini et al., 2018, 2020; De Cillà et al., 2019, 2020; Farruggio et al., 2019, 2020), as described in Supplementary File 7. The reading of wavelength was performed at 450 nm.

## *In vitro* Experiments

### Culture of Human Umbilical Vascular Endothelial Cells

Human umbilical vascular endothelial cells were purchased from ATCC (catalog no. CRL-1730™) and were maintained in Kaighn’s Modification of Ham’s F-12 Medium (F-12K Medium; ATCC; catalog no. 30-2004™), containing 2 mM L-glutamine (Euroclone S.p.A., Pero, Milan, Italy), 1500 mg/L sodium bicarbonate (Euroclone), and supplemented with 0.1 mg/mL heparin (Merck KGaA, Darmstadt; Germany), 100 μg/mL endothelial cell growth supplement (ECGS; Merck), 1% penicillin and streptomycin, and 10% fetal bovine serum (FBS; Euroclone).

### Experimental Protocol

To evaluate the effects of plasma samples taken from the elderly on cell viability (MTT Assay), mitochondrial membrane potential (JC-1 Assay) and ROS (DCFDA-Cellular ROS Detection Assay kit) on HUVEC, co-culture experiments were performed, by using specific Transwell inserts (Euroclone; Supplementary Figure 1 and Supplementary File 8). Experiments were conducted in triplicate and repeated at least three times.

### Cell Viability

Cell viability was examined in HUVEC by using the 1% 3-[4,5-dimethylthiazol-2-yl]-2,5-diphenyl tetrazolium bromide (MTT; Life Technologies Italia, Monza, Italy) dye, as previously described (Surico et al., 2017; Grossini et al., 2018; Farruggio et al., 2019, 2020; De Cillà et al., 2020). For the experiments, 200,000 HUVEC cells/well were plated in 24-Transwells plates in complete medium. HUVEC were treated with 10% plasma for 3 h, as described in Supplementary File 8. After each treatment, the medium was removed, and fresh culture medium without red phenol and FBS and with 0.5 mg/mL MTT dye was added to the 96-well plates containing the cells and incubated for 2 h at 37°C in an incubator. Thereafter, the medium was removed, and a MTT solubilization solution (dimethyl sulfoxide; DMSO; Sigma) was added and mixed in a gyratory shaker until the complete dissolution of formazan crystals. Cell viability was determined by measuring the absorbance through a spectrometer (VICTOR™ X Multilabel Plate Reader; PerkinElmer) with a wavelength of 570 nm and cell viability was calculated by setting control cells (non-treated cells), as 100%.

### Mitochondrial Membrane Potential Measurement

Mitochondrial membrane potential was measured in HUVEC with JC-1 assay (Cayman Chemical). For the experiments, 200,000 HUVEC cells/well were plated in 24-Transwells plates in complete medium and were treated as described for Cell viability. After stimulations, the medium of cells plated in starvation medium was removed and cells were incubated with 5,51,6,61-tetrachloro-1,11,3,31 tetraethylbenzimidazolyl carbocyanine iodide (JC-1) 1× diluted in Assay Buffer 1× for 15 min at 37°C in an incubator, following the manufacturer’s instruction (Cayman Chemical) and as previously performed in other cell lines (Surico et al., 2017; Grossini et al., 2018; De Cillà et al., 2020; Farruggio et al., 2020). After incubation, the cells were washed twice with Assay Buffer 1× and then the mitochondrial membrane potential was determined by measuring the red (excitation 550 nm/emission 600 nm) and green (excitation 485 nm/emission 535 nm) fluorescence through a spectrometer (VICTOR™ X Multilabel Plate Reader; PerkinElmer). The data were normalized vs control cells (non-treated cells).

### Reactive Oxygen Species Quantification

The oxidation of 2,7-dichlorodihydrofluorescein diacetate (H2DCFDA) into 2,7-dichlorodihydrofluorescein (DCF) was used to assess ROS generation, following the manufacturer’s instructions (Abcam), and as previously performed (Surico et al., 2017; Grossini et al., 2018; Farruggio et al., 2019, 2020; De Cillà et al., 2020). For the experiments, 200,000 HUVEC cells/insert were plated in 24-Transwells plates in complete medium, following the same experimental protocol followed for MTT and JC-1. Briefly, after treatments, the reactions were stopped by removing the medium and washing with PBS followed by staining with 10 μM H2DCFDA for 20 min at 37°C. The fluorescence intensity of DCF was measured at an excitation and emission wavelength of 485 and 530 nm, respectively, by using a spectrophotometer (VICTOR™ X Multilabel Plate Reader; PerkinElmer). Results were expressed as DCF fluorescence intensity, which was proportional to the amount of intracellular ROS. The data were normalized vs control cells (non-treated cells).

### Statistical Analysis

The normality of distribution of continuous variables was tested by Shapiro–Wilk test. Mean/rate ± standard deviations (SD) or confident interval (CI) were given as descriptive statistics. As concerning plasma oxidants/antioxidants and the *in vitro* experiments, all the results obtained were examined through one-way ANOVA followed by Bonferroni *post hoc* tests. The Spearman rank correlation analysis was used to investigate relationship between non-normal variables. The Mann–Whitney *U* test was used to compare non-normal numerical data among groups. A receiver operating characteristics – ROC curve analysis was performed to determinate the best cut-off values for significant biomarkers in predicting COVID-19, based on univariate analysis (the associated area under the curve – AUC, sensitivity, and specificity were also calculated). The statistical analysis was performed with SPSS for Windows version 24.0. A value of *p* < 0.05 was considered statistically significant.

## Results

The patients’ demographics at baseline are shown in Table 1. The sample included mostly women (*p* = 0.008) with a mean age of around 84 years, with a good cognitive function and a middle-severe level of dependence. Women in average significantly older than men (12 years, *p* < 0.001). Almost all the patients who did not have any contraindications to a flu vaccination have been vaccinated.

**TABLE 1:** Baseline descriptive analysis (January 2020).

Variables	Measures
**Demographics**	**Means/rates ± SD [CI 95%] (*n* = 60)**
Age (years)	84.42 ± 9.92 [81.85–86.98]
Gender, female (%)	68.3 [55.9–79]
Gender, male (%)	31.7 [21–44.1]
TBI (score)	39.88 ± 25.06 [33.41–46.36]
SPMSQ (score)	1.3 ± 0.86 [1.14–1.59]
Flu vaccination (%)	61.7 [49.1–73.2]
**Biomarkers**	
GSH (μM)	2.74 ± 0.31 [2.66–2.82]
TBARS (μM)	17.28 ± 9.94 [14.71–19.84]
8-OH-2dG (ng/mL)	45.95 ± 11.5 [42.98–48.92]
8-isoprostanes (pg/mL)	505.93 ± 157.45 [465.26–546.61]
SOD activity (%)	50.56 ± 9.56 [48.09–53.03]
25(OH) vitamin D (ng/mL)	30.29 ± 3.95 [29.27–31.31]
Thymosin β4 (ng/mL)	0.37 ± 0.12 [0.34–0.41]

*GSH, glutathione; TBARS, thiobarbituric acid reactive substances; 8-OH-2dG, 8-hydroxy 2-deoxyguanosine; SOD, superoxide dismutase; TBI, total Barthel Index; SPMSQ, Short Portable Mental Status Questionnaire.*

As also shown in Table 1, although TBARS, 8 OH-2dG and 8-isoprostanes measurements shown the presence of an “oxidative” condition in plasma of the elderly, the antioxidant system was quite preserved. Hence, vitamin D and GSH levels were almost in the physiologic range and SOD activity amounted to about 51%.

**FIGURE 1:**
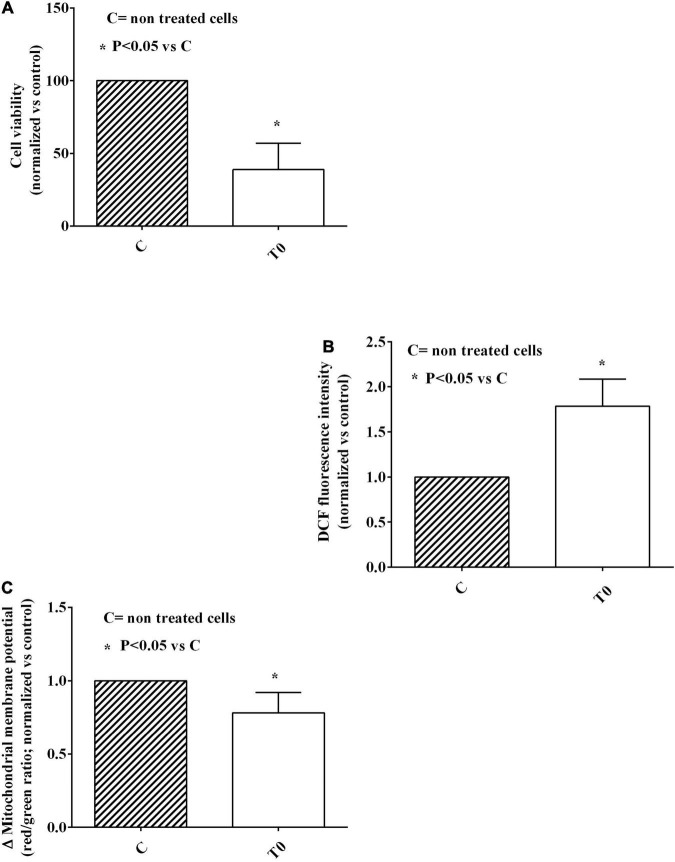
Effects of plasma of the elderly on cell viability **(A)**, reactive oxygen species (ROS; **B**) release, and mitochondrial membrane potential **(C)** in HUVEC. The results are means ± SD of three different experiments performed in triplicate. DCF, 2,7-dichlorodihydrofluorescein; T0, baseline. *Means *P* < 0.05 vs C.

It is to note that the treatment of HUVEC with plasma of the elderly was able to reduce cell viability of about 60% and to increase ROS release of about 80% in comparison with non-treated HUVEC (Figures 1A,B). In spite of this, however, mitochondrial membrane potential was quite preserved, being reduced of 20%, only (Figure 1C).

As a further descriptive analysis, we evaluated any possible association among the oxidative stress markers measured in plasma or released by HUVEC treated with plasma of the elderly (Table 2). We have observed a strong positive correlation between plasma SOD activity and 8-isoprostanes (*r* = 0.491, *p* = 0.001). We have also found a positive moderate correlation between plasma levels of Thymosin β4 and the mitochondrial membrane potential of HUVEC (*r* = 0.579, *p* = 0.001) and between the ROS released by HUVEC and plasma levels of 25(OH) vitamin D (*r* = −0.255, *p* = 0.049). Other almost significant correlations were observed: a weak negative correlation between plasma 25(OH) vitamin D and GSH, and between 25(OH) vitamin D and mitochondrial membrane potential of HUVEC, and a weak positive correlation between mitochondrial membrane potential of HUVEC and plasma 8 OH-2dG.

**TABLE 2:** Correlations among oxidative stress markers at baseline (January 2020).

Biomarkers		TBARS	8 OH-2dG	8-isoprostanes	SOD activity	25(OH) vitamin D	Cell viability	Δψ	ROS	Thymosin β4
GSH	*r*	−0.178	−0.184	−0.072	−0.100	−0.239	−0.014	−0.019	−0.065	0.115
	*p*	0.173	0.160	0.583	0.448	0.066	0.917	0.888	0.621	0.383
TBARS	*r*	1.000	−0.163	−0.139	−0.163	0.097	0.149	0.034	0.123	−0.007
	*p*		0.213	0.289	0.214	0.463	0.256	0.798	0.348	0.959
8 OH-2dG	*r*		1.000	0.106	0.166	0.142	−0.075	0.234	0.042	0.159
	*p*			0.422	0.206	0.280	0.568	0.072	0.752	0.224
8-isoprostanes	*r*			1.000	0.491**	−0.024	0.031	−0.088	−0.014	−0.082
	*p*				0.001	0.857	0.814	0.504	0.918	0.532
SOD activity	*r*				1.000	0.023	0.047	0.173	−0.164	0.096
	*p*					0.864	0.721	0.187	0.211	0.464
25(OH) vitamin D	*r*					1.000	−0.164	−0.246	0.255*	0.038
	*p*						0.211	0.058	*0.049*	0.776
Cell viability	*r*						1.000	0.147	−0.125	0.010
	*p*							0.264	0.342	0.942
Δψ	*r*							1.000	−0.178	0.579**
	*p*								0.173	0.001
ROS	*r*								1.000	0.070
	*p*									0.597

GSH, glutathione; TBARS, thiobarbituric acid reactive substances; 8-OH-2dG, 8-hydroxy 2-deoxyguanosine; SOD, superoxide dismutase; Δψ, mitochondrial membrane potential; ROS, reactive oxygen species. *p < 0.05; **p < 0.001.

The patients’ clinical outcomes are reported in Table 3. From February to April 2020, we screened clinical records for symptoms suggestive of COVID-19 and we identified 37 possible cases among 60 patients who were included in our sample at baseline. Most of the patients were affected by fever, cough, and dyspnoea, and showed low levels of blood oxygen saturation. As it has been mentioned before, the qualitative detection of nucleic acid from SARS-CoV-2 in nasopharyngeal or oropharyngeal swabs was available only from April 25th, 2020. Therefore, until the end of the study (December 31st, 2020) we identified 36 patients positive to the COVID-19 RT-PCR test among 41 patients who have been screened. A total of 19 patients died before the availability of the COVID-19 RT-PCR test and were excluded from the analysis. At the end of the observation period almost half did not survive.

**TABLE 3:** Patients clinical outcomes at follow-up (February 1st–December 31st, 2020).

Variables	Measures
Symptoms	Rates [CI 95%]
Cough (dry or with expectoration)	35 [23.9–47.5]
Rhinorrhoea and/or rhinosinusitis	3.3 [0.7–10.3]
Fever axillary temperature over 99.5°F/37.5°C	46.7 [34.4–59.2]
Blood oxygen saturation ≤ 92%	58.8 [35.6–79.3]
Diarrhea	6.7 [2.3–15.1]
Fatigue/asthenia/myalgias	5 [1.4–12.7]
Anosmia and/or ageusia	11.7 [5.4–21.5]
Dyspnoea	35 [23.9–47.5]
**Treatment (category)**	
Antibiotics (all the classes)	36.7 [25.3–49.3]
Paracetamol, dexamethasone, and/or other corticosteroids	36.7 [25.3–49.3]
Respiratory drugs and/or oxygen provision	21.7 [12.7–33.3]
Low molecular weight heparins and/or antiaggregant drugs	5 [1.4–12.7]
**Survival**	
Alive	55.0 [41.6–67.8]
Deceased	45.0 [32.1–58.4]
**Diagnosis**	
COVID-19 clinical	61.7 [48.2–73.9]
COVID-19 laboratory	87.8 [73.8–95.9]

Table 4 shows the association between the levels of oxidative stress markers taken from plasma or HUVEC treated with plasma at baseline and the patients’ outcomes. We observed statistically significant lower levels of mitochondrial membrane potential at baseline in the patients who had symptoms suggestive of COVID-19 when compared to the patients without any symptoms (mitochondrial membrane potential average values of 0.76 and 0.82, respectively; *p* = 0.035). Similar results were observed when comparing the patients who had or have not received any symptomatic therapy (*p* = 0.021). A greater difference was observed in the patients with a confirmed diagnosis of COVID-19 when compared with the elderly disease free (mitochondrial membrane potential 0.77 vs 0.86), even though such results were almost significant (*p* = 0.069).

**TABLE 4:** Association between patients’ levels of oxidative stress markers at baseline and patients’ outcomes at follow-up (Mann–Whitney *U* test, mean ± SD, significant at 0.05 level).

Biomarkers	COVID-19 clinical diagnosis (*n* = 37/60)	COVID-19 symptomatic therapy (*n* = 35/60)	COVID-19 confirmed diagnosis (*n* = 36/41)*	Patients’ overall mortality (*n* = 27/60)	COVID-19 patients’ lethality (*n* = 10/36)**
GSH (μM)	2.72 ± 0.31 (*p* = 0.538)	2.75 ± 0.33 (*p* = 0.899)	2.69 ± 0.28 (*p* = 0.381)	2.78 ± 0.35 (*p* = 0.385)	2.71 ± 0.31 (*p* = 0.849)
TBARS (μM)	17.92 ± 10.96 (*p* = 0.755)	17.95 ± 11.29 (*p* = 0.851)	17.31 ± 10.82 (*p* = 0.954)	18.77 ± 12.19 (*p* = 0.547)	20.68 ± 16.06 (*p* = 0.768)
8 OH-2dG (ng/mL)	44.44 ± 12.03 (*p* = 0.130)	44.66 ± 12.28 (*p* = 0.222)	44.37 ± 10.94 (*p* = 0.139)	46.01 ± 13.86 (*p* = 0.749)	40.09 ± 14.14 (*p* = 0.063)
8-isoprostanes (pg/mL)	514.99 ± 161.43 (*p* = 0.589)	519.56 ± 155.52 (*p* = 0.341)	502.08 ± 163.75 (*p* = 0.862)	494.97 ± 164.3 (*p* = 0.650)	481.19 ± 181.58 (*p* = 0.543)
SOD activity (%)	50.00 ± 11.00 (*p* = 0.390)	51.05 ± 10.66 (*p* = 0.840)	50.39 ± 9.81 (*p* = 0.685)	51.93 ± 10.27 (*p* = 0.588)	52.88 ± 10.86 (*p* = 0.475)
25(OH) vitamin D (ng/mL)	30.72 ± 3.66 (*p* = 0.399)	30.93 ± 3.47 (*p* = 0.222)	30.88 ± 3.98 (*p* = 0.069)	30.67 ± 4.24 (*p* = 0.577)	31.61 ± 3.49 (*p* = 0.497)
Cell viability (normalized vs control)	38.60 ± 12.29 (*p* = 0.755)	39.15 ± 12.41 (*p* = 0.851)	38.26 ± 12.32 (*p* = 0.548)	38.89 ± 11.71 (*p* = 0.923)	38 ± 13.87 (*p* = 0.590)
Δψ (normalized vs control)	0.76 ± 0.09 (*p* = 0.035)	0.76 ± 0.09 (*p* = 0.021)	0.77 ± 0.10 (*p* = 0.069)	0.78 ± 0.09 (*p* = 0.705)	0.76 ± 0.1 (*p* = 0.768)
ROS (normalized vs control)	1.77 ± 0.22 (*p* = 0.621)	1.76 ± 0.22 (*p* = 0.372)	1.77 ± 0.21 (*p* = 0.403)	1.81 ± 0.23 (*p* = 0.518)	1.73 ± 0.17 (*p* = 0.465)
Thymosin β4 (ng/mL)	0.37 ± 0.11 (*p* = 0.903)	0.37 ± 0.11 (*p* = 0.899)	0.37 ± 0.11 (*p* = 0.862)	0.37 ± 0.16 (*p* = 0.835)	0.36 ± 0.10 (*p* = 0.768)

*The patients (n = 19) who died before the execution of a molecular test were excluded from the sample. **The lethality rate was calculated including only the patients (n = 36) with a confirmed diagnosis of COVID-19 after a molecular test.

**FIGURE 2:**
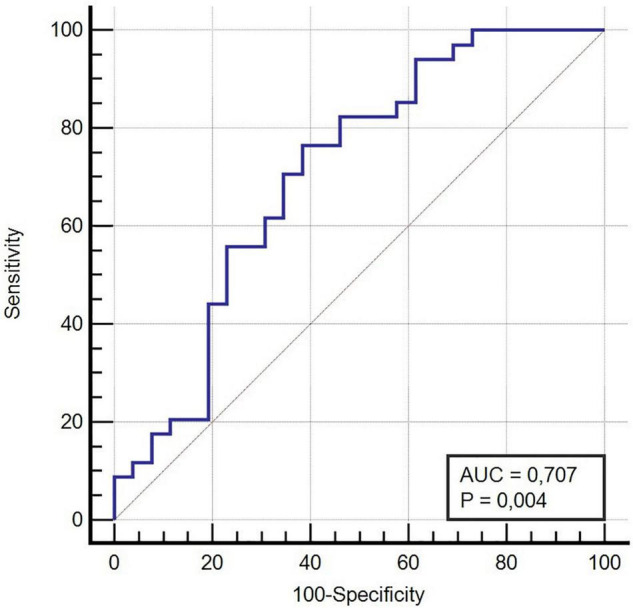
ROC Curve COVID-19 Clinical Diagnosis and mitochondrial membrane potential variation (Δψ), Area Under the Curve (AUC).

A ROC curve analysis (Figure 2) was performed to evaluate the diagnostic ability of mitochondrial membrane potential as a predictor of the future onset of COVID-19 (Figure 2). Our results showed that a threshold mitochondrial membrane potential value ≤0.79 (%) should represent the best trade-off between sensitivity (0.76, 95 CI% 0.58–0.89) and specificity (0.62, 95 CI% 0.41–0.80) when detecting the patients who could develop COVID-19 (AUC ± SE 0.71 ± 0.07).

## Discussion

The results of this study highlight the importance of mitochondrial function in the predisposition to get COVID-19 infection in a population of elderly admitted to LTC unit.

The free radical theory of aging affirms that the production of intracellular ROS represents the major determinant of life span. Moreover, it is widely accepted that oxidative stress acts as an important factor in the development of acute and chronic inflammation, and there is an association between elevated inflammatory markers and acute and chronic diseases in elderly people (Nerpin et al., 2012; Baierle et al., 2015; Kozakiewicz et al., 2019).

For the above reasons, circulating oxidative stress markers and most common antioxidants, GSH and SOD activity, were measured in plasma of elderly admitted to a residential structure.

The results obtained shown high plasma levels of TBARS, 8 OH-2dG and 8-isoprostanes. It is to note that the oxidation markers we measured are those widely acknowledged as being related to the acceleration of aging process and extensively quantified in aging studies (Pandey et al., 2010; Komosinska-Vassev et al., 2012; Wu et al., 2017; Lorenzano et al., 2018; Dzhangildinova et al., 2019).

It should also be highlighted the fact that the plasma levels of 8-isoprostanes, the products of arachidonic acid peroxidation considered as more stable, sensitive, and specific markers of oxidative stress than others (Lorenzano et al., 2018), were similar to those observed in 60–70 years old subjects performing moderate leisure time physical activity (Kozakiewicz et al., 2019).

It is also noteworthy that the increased levels of plasma oxidants were accompanied by a quite preserved plasma GSH concentration in the elderly, as well. Hence, in a previous study, plasma GSH in a population of 60–70 years old normal subjects, amounted to mean values of 2.98 μM, which was not so different from those found in our population of 80–90 years old subjects (Go and Jones, 2017).

In addition, SOD activity amounted to about 51% and the levels of vitamin D were in the normal range.

Thus, we could affirm that the plasma redox state of our population was quite “balanced.” This assumption could be confirmed by the correlation analysis performed by matching various oxidative stress markers showing positive relations between oxidants, like 8-isoprostanes, and SOD activity. Also, the results obtained by the *in vitro* experiments, in which the plasma of the elderly was used for analyzing its effect on HUVEC viability, ROS release and mitochondrial function, would confirm our hypothesis. Hence, in addition to find a reduction of cell viability and an increased ROS release, the mitochondrial function, evaluated as mitochondrial membrane potential, of HUVEC was quite preserved, as well.

As also shown for plasma oxidative stress markers, the correlation analysis performed by comparing the plasma oxidative stress markers with the results obtained in HUVEC evidenced interesting data. Indeed, a positive relation has been obtained between 8 OH-2dG and mitochondrial membrane potential, which could suggest again the existence of a balance in the redox state of the elderly, as also supported by the correlation analysis performed between 25(OH) vitamin D and ROS. Instead, the negative correlation obtained between the mitochondrial membrane potential and 25(OH) vitamin D could indicate the existence of a mutually modulation also among the “antioxidants” systems of our population.

The findings we obtained on mitochondria are of relevance. The mitochondrial membrane potential, in particular, is the central bioenergetic feature for the control of respiratory rate, ATP production and ROS generation. According to the free radical theory of aging, mitochondria could, thus, play a central role in the aging process and in the age-related diseases. Indeed, previous studies have shown that mitochondrial integrity declines as a function of age (Balaban et al., 2005).

Furthermore, the knowledge that dysfunctional mitochondria may be associated with defective immunological response to viral infections and chronic inflammation (Park and Larsson, 2011; Ren et al., 2020; Shenoy, 2020; Dominguez et al., 2021) could assume particular relevance in the context of the COVID-19 illness (Lin et al., 2006; Delgado-Roche and Mesta, 2020).

Although, so far, the precise mechanisms at the basis of COVID-19 infection have not been clearly evaluated, emerging data would interestingly suggest that the mitochondrial dysfunction may be the triggering event. Interactions of the COVID-19 virus proteins with host cell mitochondrial proteins would lead to loss of membrane integrity and cause dysfunction in the bioenergetics of the mitochondria. As a consequence, an increased ROS release; the loss of mitochondrial antiviral system, and the activation of a hyper-inflammatory state with cytokine storm, would occur (Shenoy, 2020).

It should also pointed out that changes of mitochondria function could explain the particular susceptibility of the elderly population to get a flu-like syndrome or COVID-19 infection, which may create an imbalance particularly difficult to overcome with resulting increased mortality (Shenoy, 2020).

In view of this knowledge, we have crossed data obtained about COVID-19 infection in our population with those regarding oxidative stress and, in particular, with mitochondrial function.

The results obtained in our study would confirm the above observations. Hence, in our population mitochondrial membrane potential was lower in the elderly who had symptoms suggestive of COVID-19 or with a confirmed diagnosis of COVID-19 when compared to the others.

Thus, our data would be in agreement with the knowledge that the age-related susceptibility to severe disease does not arise solely because of exposure to environmental and host risk factors, being also a consequence of maladaptive physiological processes that prejudice the ability to keep homeostasis when faced with a stressful events, which is also known as “homeostenosis.”

Overall, the results we obtained highlighted the important role played by mitochondria in aging and age-related disease. By this way the keeping mitochondrial health and effective mitochondrial reserves could be not only “prolongevity” key factors but also useful tools for resisting virus infection and for coping when the system is “stressed” (e.g., by a virus) (Moreno Fernández-Ayala et al., 2020).

Our results would, also, be in agreement with the “hormesis” theory according to which low levels of stress induce an over-compensatory response that causes positive adaptations, enabling an organism to better tolerate the stressor next time they encounter it. “Hormetic” responses could be induced by sub-lethal doses of physical activity, calorie restriction, and plant polyphenols, which would act through mitochondrial low level of stress, as a key trigger. This would result in an enhanced antioxidant capacity and a greater ability to manage the ATP/ROS when placed under stress (Nunn et al., 2020).

The problem that could arise is how to simply measure the mitochondrial function in people. About this issue, it should be highlighted that in our study the correlation analysis showed positive relation between mitochondrial membrane potential and the levels of plasma Thymosin β4, which is an anti-inflammatory hormone that can down-regulate chemokines and cytokines, as well as increase fibrinolysis (Park et al., 2021).

By this way, Thymosin β4 could represent a valuable and easily detectable marker useful as a surrogate for mitochondrial function in people, particularly, in flu-like syndromes or infections like COVID-19. In this regard it is to note that Thymosin β4 has been suggested as an off-label therapy (Romani et al., 2020).

## Conclusion

The results obtained in this study about the redox state in a population of elderly admitted to LTC unit would strengthen the importance of mitochondrial function as biomarker and target of intervention not only for aging in general but, even more, for the identification of patients that are most vulnerable to COVID-19 disease.

### Limitations

Main limitations of the present study are related to the fact that the COVID-19 RT PCR was not executed on all the subjects because not available in the first months of 2020. In addition, although the AUC value had a quite acceptable accuracy, the size of the sample under study was not aimed at this type of analysis and, therefore, it is not yet possible to recommend its application in the clinical setting on the basis of these results. In this sense, further studies will have to be carried out given the potential importance of this biomarker in the early identification of populations vulnerable to COVID-19 disease. With this in mind, it would be useful to test the effectiveness of mitochondrial membrane potential as a predictor of the development of other viral diseases. Also, no statistically significant differences were observed in patients diagnosed with COVID-19 by RT-PCR tests when compared with healthy subjects. However, the value of *p* = 0.069, which is close to the statistical significance limit, could reasonably be due to the small number of the group of healthy subjects equal to 5 people. In fact, in these subjects, the mean values of mitochondrial membrane potential amounting to 0.86, were even higher than those of 0.82 found in subjects without clinical signs and symptoms of COVID-19 or without taking symptomatic therapy. Therefore, albeit with all the necessary precautions, one could reasonably attribute the incomplete statistical significance to the effect of the loss of 19 deceased patients in the absence of a proper molecular buffer.

Moreover, other plasma oxidative stress biomarkers and parameters of mitochondrial function could have been examined. As regarding the latter, mitochondrial oxygen consumption, extracellular acidification rate, and metabolic alterations with an increase in glycolysis could be evaluated, too. The results obtained could deepen the role of COVID-19 in the modulation of cellular function (Ajaz et al., 2021).

Also, more *in vitro* experiments in HUVEC could be performed to better address the effects of a COVID-19-related milieu in the modulation of endothelial function. About this issue, the quantification of hypoxia inducible factor (HIF 1α) could improve the knowledge about the mechanism of cellular infection by COVID-19. Hence it is well known that HIF 1α can alter angiotensin converting enzyme 1 and 2 expression and by this way it could modulate the COVID-19 entry within cells (Gheblawi et al., 2020).

It should also be noteworthy that in our study we did not measure the blood oxygenation rate and did not evaluate oxygen binding assessment, which would be mandatory in case of COVID-19 infection. This was due to the fact that our initial purpose was to perform a study about aging and we could not imagine that the COVID-19 infection would break out in the following months. For the same reason we did not evaluate any hemolytic variable.

Finally, the deepening of which could be the circulating factor(s) involved in endothelial cell dysfunction should be mandatory. The acquired information about this issue could assume clinical relevance as early diagnostic or prognostic biomarker useful to identify high “risk” patients for the development of age-related disease and infections.

## Data Availability Statement

The original contributions presented in the study are included in the article/Supplementary Material, further inquiries can be directed to the corresponding author/s.

## Ethics Statement

The studies involving human participants were reviewed and approved by the Ethical Committee of the “Azienda Ospedaliera Maggiore della Carità” University Hospital in Novara. The patients/participants provided their written informed consent to participate in this study.

## Author Contributions

EG, CR, PZ, CG, and MP: conceptualization and funding acquisition. DC, DG, SR, and SK: methodology and investigation. EG, CR, PZ, CG, and MP: resources. EG, DC, CR, DG, SK, and MP: formal analysis. EG, DC, CR, SR, DG, PZ, CG, SK, and MP: supervision, validation, and visualization. EG, DC, SK, and MP: writing – original draft preparation. EG, DC, CR, SR, DG, PZ, CG, SK, and MP: writing – review and editing. All authors were involved in editing the manuscript and had final approval of the submitted and published versions.

## Conflict of Interest

The authors declare that the research was conducted in the absence of any commercial or financial relationships that could be construed as a potential conflict of interest.

## Publisher’s Note

All claims expressed in this article are solely those of the authors and do not necessarily represent those of their affiliated organizations, or those of the publisher, the editors and the reviewers. Any product that may be evaluated in this article, or claim that may be made by its manufacturer, is not guaranteed or endorsed by the publisher.
