# Supplementary material for: Association Between Plasma Redox State/Mitochondria Function and a Flu-Like Syndrome/COVID-19 in the Elderly Admitted to a Long-Term Care Unit
Source: Front Physiol. 2021 Dec 15;12:707587. doi: 10.3389/fphys.2021.707587 (PMC8715756; doi:10.3389/fphys.2021.707587)
Supplement: Supplementary file 1 [file Data_Sheet_1.docx]

***Supplementary materials***

***Supplementary File S1***

***GSH quantification***

Plasma GSH measurement was performed by using the Glutathione Assay Kit (Cayman Chemical, Ann Arbor, MI, USA), as previously described (De Cillà et al., 2019; Farruggio et al., 2019; Grossini et al., 2020). For the experiments, each plasma sample was deproteinated adding an equal volume of MPA solution to the sample that was centrifuged at 2000 g for 2 min. Thereafter, the supernatant was collected and 50 μl/ml of TEAM reagent was added to each sample in order to increase the pH. Fifty μl of the samples was transferred to a 96‐well plate where GSH was detected following the manufacturer's instructions through a spectrophotometer (VICTOR™ X Multilabel Plate Reader), at excitation/emission wavelengths of 405-414 nm. To ensure accurate GSH quantification (as µM), a reference curve with the GSH Standard was prepared.

***Supplementary File S2***

***TBARS quantification***

Plasma TBARS were determined as malonyldialdeide (MDA) release. MDA measurement was performed by using the TBARS assay Kit (Cayman Chemical), as previously performed (De Cillà et al., 2019; Surico et al., 2019; Grossini et al., 2020). For the experiments, 100 µl of each plasma sample was added to 100 µl of sodium dodecyl sulfate (SDS) solution and 2 ml of the Color Reagent, following the manufacturer's instruction. Each sample was boiled for 1 h and then transferred on ice for 10 min in order to stop the reaction. After this time, each sample was centrifuged for 10 min at 1600 g at 4°C and then, 150 μl was transferred to a 96‐well plate where MDA was detected following the manufacturer's instructions through a spectrophotometer (VICTOR™ X Multilabel Plate Reader), at excitation/emission wavelengths of 530–540 nm. In order to quantify the correct value of TBARS in each sample (expressed as MDA in µM), a reference standard curve with the TBARS Standard was prepared.

**Supplementary File S3**

***8-hydroxy 2 deoxyguanosine (8 OH-2dG) quantification***

Plasma 8 OH-2dG measurement was performed by using the 8-hydroxy 2 deoxyguanosine ELISA Kit (Abcam) (Surico et al., 2017, 2019; Grossini et al., 2018, 2020; De Cillà et al., 2019, 2020; Farruggio et al., 2019, 2020). For the experiments, 50 μl of each plasma sample and standard was added to each well. Fifty μl of 8-hydroxy 2 deoxyguanosine Antibody Preparation was added to each well and then, the plate was incubated at room temperature (RT) for 1 h. After 1 h, each well was washed 4 times with 300 μl of 1X Wash Buffer and then, 100 μl of Substrate Solution (tetramethyl-benzidine; TMB) was added to each well and incubated for 30 min at RT in the dark. The enzyme reaction was stopped by adding 100 μl of Stop Solution (1 M phosphoric acid) into each well and plate was read immediately. The 8 OH-2dG was detected following the manufacturer's instructions through a spectrophotometer (VICTOR™ X Multilabel Plate Reader), using a wavelength of 450 nm. The value of each sample (ng/ml) was quantified in respect to 8-hydroxy 2 deoxyguanosine standard curve.

***Supplementary File S4***

***8 isoprostanes quantification***

Plasma 8 isoprostanes (F2 isoprostanes) measurement was performed by using the 8 isoprostanes ELISA Kit (Abcam) (Surico et al., 2017, 2019; Grossini et al., 2018, 2020; De Cillà et al., 2019, 2020; Farruggio et al., 2019, 2020). For the experiments, in order to isolate and purify 8 isoprostanes from each plasma sample, 1 ml of plasma (adjusted with 12 μl acetic acid to pH 4) and 1 ml of ethyl acetate were combined and centrifuged at 2000 rpm for 10 min at RT. By this way three phases were formed: the upper organic phase (ethyl acetate phase containing lipoproteins), the interphase (containing proteins) and a lower phase (aqueous phase). The organic phase was collected and transfer in a new tube. The interphase was discarded and the lower phase was transferred in a new tube and used for repeating the acetate extraction for three times. Subsequently, the organic phase was evaporated in a Speedvac. The dried residues were dissolved in 2 ml of 20% KOH solution and incubated for 1 h, at 50 °C. The 2 ml of the aqueous solution were diluted with 3 ml of H_2_O and pH was adjusted using 20% formic acid. Two ml of ethyl acetate was added and centrifuged at 2000 rpm for 10 min at RT. The procedure was repeated for three times. The ethyl acetate in the upper phase was dried in a Speedvac. For the ELISA assay, the dried sample-residue was dissolved in 20 μl ethanol and 130 μl of 1X Sample Dilution Buffer. For the competitive 8 isoprostanes ELISA assay, 150 μl of sample was further diluted 1:16 with a final pH 7,4. At the end of samples preparation, 200 μl of 1X Sample Dilution Buffer was added into the blank wells, while 100 μl of 1X Sample Dilution Buffer was added into maximum binding control wells. One hundred μl of each sample and Standard were added into appropriate wells. Furthermore, 100 μl of the 1X-HRP conjugate was added in each well, except in the blank control wells. The plate was incubated at RT for 2 h. After this time, the plate was washed 3 times with 400 μl of 1X Wash Buffer and then, 200 μl of TMB was added to all of the wells and incubated for 30 min at RT. The enzyme reaction was stopped by adding 50 μl of Stop Solution (2 N sulfuric acid) and plate was read immediately. The 8 isoprostanes was detected following the manufacturer's instructions through a spectrophotometer (VICTOR™ X Multilabel Plate Reader), using a wavelength of 450 nm. The value of each sample (pg/ml) was quantified in respect to 8 isoprostanes standard curve.

***Supplementary File S5***

***25(OH) Vitamin D quantification***

Plasma 25(OH) Vitamin D was measured by using the 25(OH) Vitamin D ELISA Kit (Abcam) (Surico et al., 2017, 2019; Grossini et al., 2018, 2020; De Cillà et al., 2019, 2020; Farruggio et al., 2019, 2020). For the experiments, 90 μl of the Dissociation Buffer was added to each well. Ten μl of Sample Diluent was added to B0 wells (Maximum binding) and NSB (Non-Specific Binding) wells. Ten μl of plasma sample (diluted 1:10) and Standard were added to the appropriate wells with Dissociation Buffer. The plate was incubated for 5 min on a shaker at RT. After this time, 50 μl of 1X Conjugate was added to each well, 50 μl of the Conjugate Diluent was added to NSB wells and, finally, 50 μl of the supplied Antibody was added to each well, except in the NSB wells. The plate was incubated for 1 h on a shaker at RT. After 1 h, 200 μl of p-NitroPhenyl Phosphate (pNpp), the chromogenic substrate for the detection of alkaline phosphatase (AP), was added to each well and the plate incubated for 30 min on a shaker at RT. The enzyme reaction was stopped by adding 50 μl of Stop Solution (1 M phosphoric acid) into each well and plate was read immediately through a spectrophotometer (VICTOR™ X Multilabel Plate Reader), using a wavelength of 405 nm. The value of each sample (ng/ml) was quantified in respect to 25(OH) Vitamin D standard curve.

***Supplementary File S6***

***SOD activity***

Total SOD activity was determined in plasma by using the Superoxide Dismutase Activity Assay Kit (Abcam) (Surico et al., 2017, 2019; Grossini et al., 2018, 2020; De Cillà et al., 2019, 2020; Farruggio et al., 2019, 2020). For the experiments, 20 μl of each plasma sample (diluted 1:5) or standard were added in each well, together with 200 μl of WST working Solution and 20 μl of Enzyme Working Solution. In the Blank 1 well, 10 μl of double-distilled water (ddH_2_O), 200 μl of WST working Solution and 20 μl of Enzyme Working Solution were added. In the Blank 2 well, 20 μl of each plasma sample (diluted 1:5) or standard were added in each well, together with 200 μl of WST working Solution and 20 μl of Dilution Buffer. Finally, in the Blank 3, 20 μl of ddH_2_O, 200 μl of WST working Solution and 20 μl of Dilution Buffer, were added. The plate was left on a shaker for 5 min and then, 20 μl of Stop solution was added in each well. After incubation at 37°C for 20 min, the fluorescence was read by using a spectrophotometer (VICTOR™ X Multilabel Plate Reader), with a wavelength of 450 nm. In order to quantify SOD activity (%), a reference standard value prepared by using xanthine oxidase was used.

**Supplementary File S7**

**Thymosin β4**

Thymosin β4 (Human TMSβ4) was measured by using the Thymosin beta 4 (Human TMSβ4) ELISA Kit (FineTest; Wuhan Fine Biotech Co.; Wuhan, China) (Surico et al., 2017, 2019; Grossini et al., 2018, 2020; De Cillà et al., 2019, 2020; Farruggio et al., 2019, 2020). Before the assay the dilution of the samples was done accordingly with the provided sample diluent and plate was washed 5 times. For the experiments, 50 μl of the standard, blank or sample were added to appropriate wells. The blank was added with 50 μl sample/dilution buffer. Then 50 μl of Biotion-labeled Antibody Working solution was added to each well immediately and incubated for 45 min at 37°C. Then the plate was washed 3 times with washing buffer. 100 μl of HRP-Streptavidin Conjugate (SABC) was added to each well and incubated for 30 min at 37°C. Again, washing of plate was carried out for 5 times with washing buffer. 90 μl of TMB Substrate was then added to each well and incubated for 10-20 min in dark at 37°C. Thereafter, 50 μl of stop solution was added to each well and the fluorescence was read by using a spectrophotometer (VICTOR™ X Multilabel Plate Reader), with a wavelength of 450 nm. Thymosin β4 was expressed as ng/ml.

***Supplementary File S8***

***Experimental protocol***

To evaluate the effects of plasma samples taken from the elderly on cell viability (MTT Assay), mitochondrial membrane potential (JC-1 Assay) and total ROS (DCFDA-Cellular ROS Detection Assay kit) on HUVEC, co-culture experiments were performed by using specific Transwell inserts (Supplementary Figure 1).

These inserts are permeable supports that permit cells to uptake and secrete molecules on both their basal and apical surfaces and thereby carry out metabolic factors in a more natural fashion.

For the experiments, plasma samples were plated in the apical compartment of the insert and left to act for 3 h, while, HUVEC were plated in the basal compartment. Experiments were performed with 10% plasma calculated in relation with total volume of each insert. Some cell samples were not treated with plasma and were used as control. After 3 h stimulation with plasma, the inserts were removed and various assays were performed in triplicate and analyzed by using a spectrophotometer.


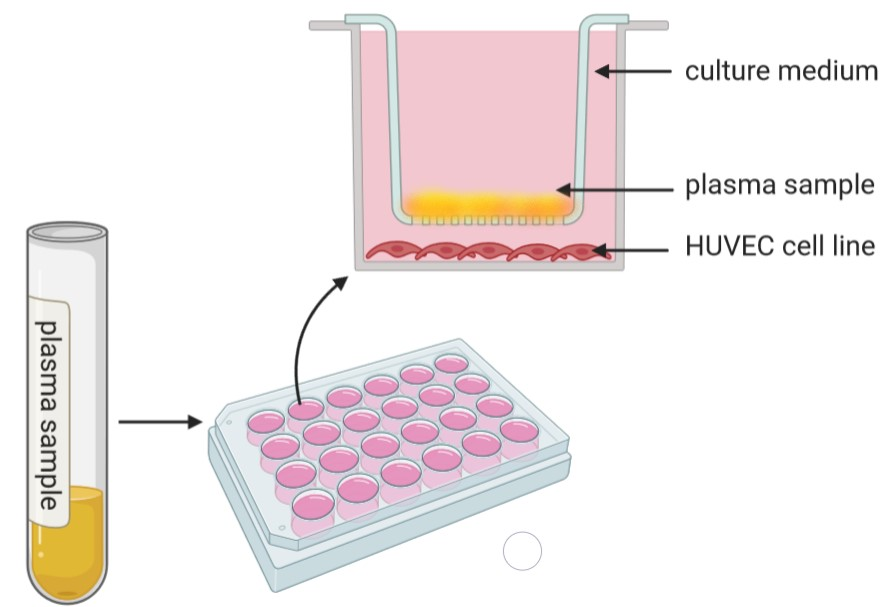


Supplementary Figure 1. Transwell inserts

**References**

De Cillà, S., Farruggio, S., Cocomazzi, G., Mary, D., Alkabes, M., Rossetti, L., et al. (2020). Aflibercept and Ranibizumab Modulate Retinal Pigment Epithelial Cells Function by Acting on Their Cross Talk with Vascular Endothelial Cells. Cell. Physiol. Biochem. 54, 161–179. doi:10.33594/000000212.

De Cillà, S., Vezzola, D., Farruggio, S., Vujosevic, S., Clemente, N., Raina, G., et al. (2019). The subthreshold micropulse laser treatment of the retina restores the oxidant/antioxidant balance and counteracts programmed forms of cell death in the mice eyes. Acta Ophthalmol. 97, e559–e567. doi:10.1111/aos.13995.

Farruggio, S., Cocomazzi, G., Marotta, P., Romito, R., Surico, D., Calamita, G., et al. (2020). Genistein and 17β-Estradiol Protect Hepatocytes from Fatty Degeneration by Mechanisms Involving Mitochondria, Inflammasome and Kinases Activation. Cell. Physiol. Biochem. 54, 401–416. doi:10.33594/000000227.

Farruggio, S., Raina, G., Cocomazzi, G., Librasi, C., Mary, D., Gentilli, S., et al. (2019). Genistein improves viability, proliferation and mitochondrial function of cardiomyoblasts cultured in physiologic and peroxidative conditions. Int. J. Mol. Med. doi:10.3892/ijmm.2019.4365.

Grossini, E., Farruggio, S., Pierelli, D., Bolzani, V., Rossi, L., Pollesello, P., et al. (2020). Levosimendan Improves Oxidative Balance in Cardiogenic Shock/Low Cardiac Output Patients. J. Clin. Med. 9, 373. doi:10.3390/jcm9020373.

Grossini, E., Farruggio, S., Raina, G., Mary, D., Deiro, G., and Gentilli, S. (2018). Effects of Genistein on Differentiation and Viability of Human Visceral Adipocytes. Nutrients 10, 978. doi:10.3390/nu10080978.

Surico, D., Bordino, V., Cantaluppi, V., Mary, D., Gentilli, S., Oldani, A., et al. (2019). Preeclampsia and intrauterine growth restriction: Role of human umbilical cord mesenchymal stem cells-trophoblast cross-talk. PLoS One 14, e0218437. doi:10.1371/journal.pone.0218437.

Surico, D., Ercoli, A., Farruggio, S., Raina, G., Filippini, D., Mary, D., et al. (2017). Modulation of Oxidative Stress by 17 β-Estradiol and Genistein in Human Hepatic Cell Lines In Vitro. Cell. Physiol. Biochem. 42, 1051–1062. doi:10.1159/000478752.
